# Synergy quality assessment of muscle modules for determining learning performance using a realistic musculoskeletal model

**DOI:** 10.3389/fncom.2024.1355855

**Published:** 2024-05-30

**Authors:** Akito Fukunishi, Kyo Kutsuzawa, Dai Owaki, Mitsuhiro Hayashibe

**Affiliations:** Department of Robotics, Graduate School of Engineering, Tohoku University, Sendai, Japan

**Keywords:** muscle synergy, redundancy, neural network, optimization, motor control

## Abstract

How our central nervous system efficiently controls our complex musculoskeletal system is still debated. The muscle synergy hypothesis is proposed to simplify this complex system by assuming the existence of functional neural modules that coordinate several muscles. Modularity based on muscle synergies can facilitate motor learning without compromising task performance. However, the effectiveness of modularity in motor control remains debated. This ambiguity can, in part, stem from overlooking that the performance of modularity depends on the mechanical aspects of modules of interest, such as the torque the modules exert. To address this issue, this study introduces two criteria to evaluate the quality of module sets based on commonly used performance metrics in motor learning studies: the accuracy of torque production and learning speed. One evaluates the regularity in the direction of mechanical torque the modules exert, while the other evaluates the evenness of its magnitude. For verification of our criteria, we simulated motor learning of torque production tasks in a realistic musculoskeletal system of the upper arm using feed-forward neural networks while changing the control conditions. We found that the proposed criteria successfully explain the tendency of learning performance in various control conditions. These result suggest that regularity in the direction of and evenness in magnitude of mechanical torque of utilized modules are significant factor for determining learning performance. Although the criteria were originally conceived for an error-based learning scheme, the approach to pursue which set of modules is better for motor control can have significant implications in other studies of modularity in general.

## 1 Introduction

Modularity is believed to simplify control by assuming functionally coordinated modules in the control architecture instead of controlling all control units (d'Avella et al., 2015) and is considered to serve to control redundant motor systems effectively. Among the various forms of modularity that have been proposed (Rückert and d'Avella, 2013; Sartori et al., 2013; Sternad et al., 2013; Alessandro et al., 2014; Hayashibe and Shimoda, 2014), muscle synergy hypothesis is characterized by a particularly small number of combinations of muscle coordination patterns. The muscle synergy hypothesis is gaining phenomenological support based on previous studies (d'Avella and Bizzi, 2005; Overduin et al., 2008; Roh et al., 2012; Hilt et al., 2018) and its neural implementation is being identified (Giszter et al., 1993; Kargo et al., 2010; Roh et al., 2011; Takei et al., 2017). Following the rudimentary but successful demonstration of the muscle synergy hypothesis, modularity is gaining popularity not only in neuroscience but also in various fields such as robotics and rehabilitation (d'Avella et al., 2015; Ting et al., 2015; Brock and Valero-Cuevas, 2016).

In line with this, researchers started to study what effects the central nervous system (CNS) would have in adopting modularity in motor control rather than full-rank control. The typical aspects regarded as advantages of modularity are its facilitating effects of motor learning or planning (Rückert and d'Avella, 2013; Diamond and Holland, 2014; Hagio and Kouzaki, 2018; Al Borno et al., 2020; Chen and Qiao, 2020; Berg et al., 2023) and partial generalization capability (Rückert and d'Avella, 2013; Taïx et al., 2013; Al Borno et al., 2020; Chen and Qiao, 2020; Kutsuzawa and Hayashibe, 2022; Berg et al., 2023). These aspects will provide evolutionary benefits to organisms because combining a limited number of learned repertoires for realizing a new motor skill is more efficient than an exhaustive search in full-rank motor space for an optimal solution if the generated motor skill is enough (Loeb, 2021).

On the other hand, the effectiveness of modularity in motor control is still controversial. The controversial point is whether a synergistic controller performs practically enough compared with an optimal control or optimization. Due to a potential performance limitation of modularity deriving from its low dimensionality, researchers had pushed modularity off to an extreme position relative to the optimal control or optimization (Hirashima and Oya, 2016; Berret et al., 2019). To fill the gap between these two positions, researchers have tested the performance of modularity compared with the optimized one (Berniker et al., 2009; McKay and Ting, 2012; de Rugy et al., 2013; Inouye and Valero-Cuevas, 2016; Al Borno et al., 2020). However, the studies have reported conflicting results; Some researchers suggested that the degradation in performance is negligible (Berniker et al., 2009; Al Borno et al., 2020), while others suggested that degradation matters significantly (McKay and Ting, 2012; de Rugy et al., 2013; Inouye and Valero-Cuevas, 2016). It is necessary to locate what makes the disputes over modularity and optimality complicated.

We speculate that one of the causes of these complexities in modularity comes from the confusion of the performance of the specific modules of interest with the overall discussion of the performance of modularity. There is no doubt that the effectiveness of modularity can be affected by module extraction algorithms, complexities of a musculoskeletal system, control policy, and even tasks. For instance, Hagio *et al*. observed that the control performance and effect of learning facilitation of modularity depends on the mechanical properties of modules (Hagio and Kouzaki, 2018). Similarly, Borno *et al*. pointed out that the complexities in the musculoskeletal model could affect the effectiveness of modularity (Al Borno et al., 2020). They found that the degradation of learning performance of modularity compared with individual muscle control is slight when a realistic musculoskeletal model is used. Furthermore, they also reported that control performance variance depends on the module extraction algorithms. As for generalization, Kutsuzawa *et al*. found that the successful generalization capability of modularity is provided by modules extracted by multiple tasks, whereas modularity with modules extracted by a single task fails to generalize (Kutsuzawa and Hayashibe, 2022). Their results support that the mechanical repertoire of modules, rather than the number of modules, is significant for motor generalization. However, except for these studies, most previous research has not paid much attention to such module-dependent variation in the performance of modularity, which can be considered the cause of controversial arguments concerning modularity. Therefore, it is necessary to quantitatively clarify what are the essential factors of modules that determine the performances and provide a better understanding of modularity.

**Figure 1:**
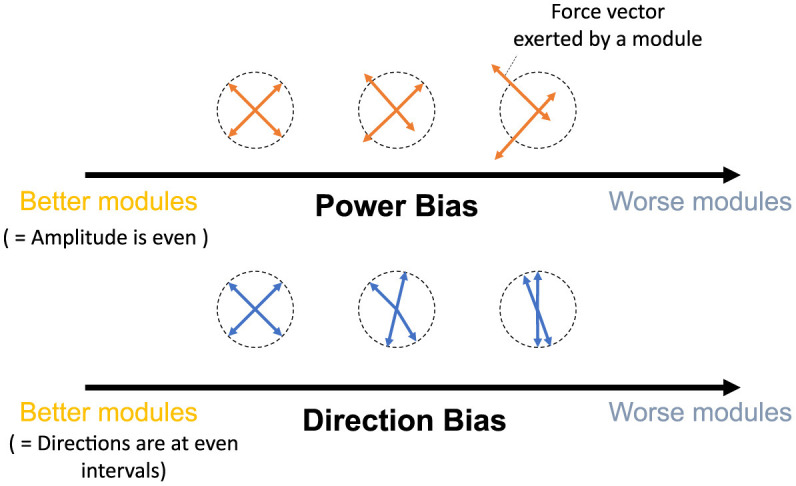
Criteria proposed by this study. The yellow and blue arrows represent the torque exerted by the utilized modules, while the dotted circle signifies the normalized amplitude of the torque. Power Bias quantifies the unevenness of torque amplitude (**top**), and Direction Bias quantifies the irregularity in torque direction (**bottom**) of the modules in task space, respectively. These criteria assess modules with lower bias as superior, indicating better motor performance. Conversely, modules with higher bias are predicted to result in inferior motor performance.

This study aims to provide refined insights into modularity by quantitatively associating the relationship between the performance/effect of modularity and utilized modules. To this end, we quantify the modules with their mechanical properties inspired by the previous studies (Hagio and Kouzaki, 2018). Then, we verified the relationship between them and the popular measure of modularity: task accuracy and learning speed in error-based learning. In concrete, we proposed two criteria (Figure 1), Direction Bias (DB) and Power Bias (PB), to quantify the modules. DB quantifies the unevenness of the directions of the torque vectors exerted by individual modules, whereas PB quantifies the unevenness of the amplitude of the torque vectors. These metrics were developed by adding heuristics to theoretical predictions regarding the convergence speed and accuracy of learning in gradient-based learning of linear neural networks to make them applicable to more realistic situations. Specifically, we designed DB and PB to predict the learning speed and accuracy of isometric control of a realistic musculoskeletal system of a non-linear neural network with non-negative constraints for muscle actuation. Our metrics could reveal the relationship between the realistic musculoskeletal learning system and the mechanical function of muscles or muscle synergies, advancing our understanding of motor control and muscle synergies. To validate the criteria, we did two separate experiments: (1) Similar to the conventional studies, we compared the task accuracy and learning speed of the independent muscle controller and the muscle synergy controller; and (2) We verified the explanation capabilities of criteria for task accuracy and learning speed by providing various sets of possible modules, changing the number of modules, or scaling the passive force elements. Our analysis demonstrates that muscle synergies should be formed in a way that could effectively compensate for the musculoskeletal complexities to improve the performance of the modularity. Our approach to quantitative module assessment can have significant implications in other studies of modularity.

## 2 Materials and methods

### 2.1 Musculoskeletal plant

In this study, in contrast with the simplified musculoskeletal plants (Hirashima and Nozaki, 2012; Hagio and Kouzaki, 2018; Song et al., 2022), we implemented a more realistic musculoskeletal plant for an isometric force production task by approximating the musculoskeletal dynamics calculation of OpenSim (Delp et al., 2007) so as not to miss the significant properties of musculotendon dynamics such as the passive force element and the force-length relationships of the muscles. In this study, we choose a realistic upper limb musculoskeletal model that we approximate and implement as an affine function of muscle activation (Saul et al., 2015).

The *n*-th muscle's force, *F*_*n*_, is given by multiplication of its maximum isometric force, *F*_*iso*_*n*__, normalized force function, *f*_*n*_(*a, l*_*n*_), and cosine of pennation angle, ψ_*n*_(*l*_*n*_) (Equation 1). Additionally, we can give the normalized force coefficient, *f*_*n*_(*a, l*_*n*_), as a summation of the active force element, *f*_*AL*_*n*__(*l*_*n*_), that is scaled by muscle activation, *a*_*n*_, and the passive force element, *f*_*PL*_*n*__(θ) (Equation 2). This is uniquely determined by the length of the muscle fiber, *l*_*n*_, when enough time for muscle activation and muscle fiber speed convergence has passed.


(1) $$\begin{array}{l} F_{n}=F_{iso_{n}}f_{n}\left(a_{n},l_{n}\right)cos\left(\psi_{n}\left(l_{n}\right)\right) \end{array}$$

(2) $$\begin{array}{l} f_{n}\left(a_{n},l_{n}\right)=a_{n}f_{AL}\left(l_{n}\right)+f_{PL}\left(l_{n}\right) \end{array}$$

In the musculoskeletal computation framework of OpenSim, the fiber length, *l*_*n*_, is searched so that the tendon force and the fiber force, *F*_*iso*_*n*__*f*_*n*_(*a*_*n*_, *l*_*n*_)*cos*(α_*n*_), are in equilibrium under a constraint that the total length of them is identical to the muscle-tendon length calculated from the musculoskeletal geometry, i.e., the posture of the model, θ. In this study, the posture is given as 2D vectors (θ ∈ ℝ^2^), with components representing the normalized values of shoulder horizontal flexion/extension angles and elbow flexion/extension angles. We normalized the posture value so that it falls within the interval of (0, 1). Furthermore, because the fiber force depends on muscle activation *a*_*n*_(Equation 2), the fiber length, *l*_*n*_, is affected by muscle activation. Therefore, the fiber length is determined by the model's posture and the activation at convergence, *l*_*n*_ : = *l*_*n*_(θ, *a*_*n*_), indicating that the muscle force is a function of the musculoskeletal posture and the activation of the muscle. However, it is difficult to find the analytical solution of fiber length directly, and active/passive force–length relationship computation requires large computation costs. Hence, we approximate the muscle force as a first-order form of the muscle activation determined by the model's posture (Equation 3).


(3) $$\begin{array}{l} F_{n}\approx F_{iso_{n}}\left(a_{n}k_{AL_{n}}\left(\theta \right)+k_{PL_{n}}\left(\theta \right)\right) \end{array}$$

Where *F*_*iso*_*n*__ denotes the maximum isometric force of *n* th muscle. We found the active force coefficient, *k*_*AL*_*n*__(θ), and the passive force coefficient, *k*_*PL*_*n*__(θ), by applying a least-square matching to the relation map of the muscle activation and the muscle force. We extracted 20 samples of the muscle force by intervals of Δ*a*_*n*_ = 0.05 while fixing the posture of the musculoskeletal model. In this research, we approximated the mechanics of the musculoskeletal model at three postures that we refer to as “Base Posture,” “Fully Extended,” and “Mildly Extended” (see below and Section 2.4.1), and we conducted this sampling for each posture.

**Figure 2:**
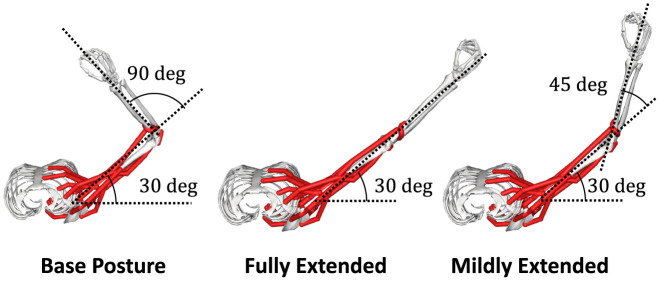
Postures of interest. In the posture of the musculoskeletal model, which we refer to as “Base Posture”, the shoulder is horizontally flexed at a 30-degree, and the elbow is flexed at a 90-degree (**left**). In the other two postures, which we refer to as “Fully Extended” (**left**) and “Mildly Extended” (**right**), respectively, the elbow is fully or mildly extended from Base Posture.

**Figure 3:**
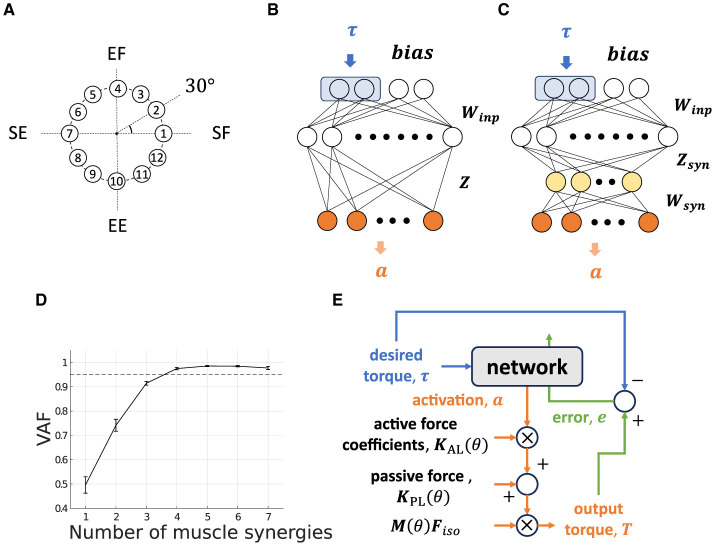
Non-synergy and synergy model for isometric shoulder and elbow torque production task. **(A)** Twelve torque targets are uniformly distributed on a circumference with a radius of 1[Nm] at 30-degree intervals in the torque space. **(B, C)** Neural network models without modules [**(B)** “Non-synergy model”] and with modules [**(C)** “Synergy model”]. Two neurons at the first layer surrounded by a blue rectangle receive a desired torque target, and adjacent neurons receive task-irrelevant bias neurons. The yellow-colored neurons in the third layer in the synergy model represent muscle synergy. The gray-colored neuron in the final layer represents muscle activation neurons. **(D)** Variance Accounted For (VAF) for determining the number of muscle synergies. The dashed line represents VAF = 0.95. **(E)** Flowchart of the network output and update.

We choose a two-joint torque production system for the task: shoulder horizontal flexion/extension and elbow flexion/extension, denoted as “elv angle” and “elbow flexion”, respectively, in the model. In this research. we decide the task posture θ_*task*_ of the musculoskeletal model, which we refer to as “Base Posture” by horizontally and vertically flexing the shoulder by 30 degrees and 90 degrees, respectively, and flexing the elbow by 90 degrees (Figure 2). Similarly to the previous study (Hagio and Kouzaki, 2018), the target torque combination was randomly chosen from the 12 ideal torque targets. The 12 torque targets are evenly distributed along the circumference of the circle with a radius of 1[Nm] in the torque space (Figure 3A). The resultant torque, ***T***, was calculated by multiplication of the moment arm matrix of *N*_*mus*_ simulated muscles, $M\left(\theta \right)\in {\mathbb{R}}^{2\times N_{mus}}$, and muscle force vectors, $F={\left[F_{1},F_{2},...,F_{N_{mus}}\right]}^{⊤}$ (Equation 4).


(4) $$\begin{array}{l} T=M\left(\theta \right)F_{iso}\left(K_{AL}\left(\theta \right)a+K_{PL}\left(\theta \right)\right) \end{array}$$

(5) $$\begin{array}{l} =T_{AL}+T_{PL} \end{array}$$

Where ***K***^*AL*^ and ***F***_*iso*_ denote diagonal matrices whose diagonal components are the active force coefficients and the maximum isometric force, respectively, and ***K***^*PL*^ denotes a ${\mathbb{R}}^{N_{mus}}$ vector of the passive force. $a\in {\mathbb{R}}^{N_{mus}}$ denotes the vector of the muscle activation. The mechanical function of the muscles or modules, which is defined as a mechanical pulling vector, is calculated as an active force component of Equation (4), ***M***(θ)***F***_*iso*_***K***_*AL*_***a***. These torque calculation procedures are summarized as orange-colored flows in Figure 3E.

In this research, we extracted 22 task-relevant muscles (*N*_*mus*_ = 22, deltoid anterior [DeltAnt], deltoid middle [DeltMid], deltoid posterior [DeltPst], supraspinatus, infraspinatus, subscapularis, teres minor [TerMin], teres major [TerMaj], pectoralis major clavicular [PecMajClv], pectoralis major sternal [PecMajStr], pectoralis major ribs [PectMajRib], latissmus dorsi thoracic [LatDorTho], latissmus dorsi lumbar [LatDorLum], latissmus dorsi iliac [LatDorIil], coracobrachialis, triceps long [TRILong], triceps lateral [TRILat], triceps medial [TRIMed], biceps long [BICLong], biceps short [BICShort], brachialis, and brachioradialis), which are agonists or antagonists of the joints movement.

### 2.2 Neural network models

We are primarily focused on examining how the mechanical properties of a set of modules influence the performance of modularity in error-based learning. To this end, similarly to the related works, we modeled the motor learning system as a descending feed-forward neural network. Although a feed-forward neural network is a largely simplified motor control system which ignores some important complexities of actual neural networks, such as sensory feedback loop and recurrent connection, it provides powerful description capabilities against data of experimentally observed neural behavior (Rokni et al., 2007; Hirashima and Nozaki, 2012; Takiyama and Okada, 2012; Song et al., 2022). In this research, we modeled two types of neural network (Figures 3B, C): one is a non-synergy model that the CNS directly and individually control muscles, and the other is synergy model that the CNS indirectly controls muscles via a small number of modules. The performance of the network is defined as the learning error at the final learning trials and its learning speed. The function of the network and how it learns are summarized as blue-colored and green-colored flows in Figure 3E.

#### 2.2.1 Non-synergy model

Our network's structure is the same as the one used in previous studies (Hirashima and Nozaki, 2012; Hagio and Kouzaki, 2018; Song et al., 2022), with the exception of the addition of two bias neurons at the input layer (Figure 3B). Bias neurons, ***b***, are non-zero constant vectors that have no correlation with the task. This simplified bias neuron addition can be partially justified because the neurons of the primary motor cortex are active for various motor parameters (Scott, 2008; Shenoy et al., 2013; Gallego et al., 2018) rather than specialized for specific parameters such as gain of the task, and there should be neurons which shows almost no correlation with the task. In our musculoskeletal model, we added the passive force component that makes an input–output relationship of the plant affine transformed one (Equation 3) to reproduce the OpenSim musculoskeletal dynamics, and added bias neurons serve to compensate for this disturbance. The input layer contains two torque-active neurons that receive ideal torque, ***τ*** randomly from the 12 torque production targets (Figure 3A). The other part of the network, e.g., the first intermediate layer (M1 layer), which models the primary motor cortex and consists of 1000 neurons, computes and learns required motor commands in accordance with the input weight matrix, ***W*****_*inp*_**. The innervation matrix, ***Z***, which models the spinal cord, computes the resulting muscle activation vector, ***a***;


(6) $$\begin{array}{l} a=\left\lfloor ZW_{inp}x\right\rfloor \end{array}$$

Where ***x*** denotes an input vector, which is a concatenated vector of desired torque and bias (Equation 7).


(7) $$\begin{array}{l} x=\left(\begin{array}{c} \tau \\ b \end{array}\right) \end{array}$$

In a non-linear network, the muscle activation, ***a***, is given by filtering the output of the network ***ZW*****_*inp*_*x*** with non-negative constraints ⌊·⌋. We initialized the innervation matrix and input weight matrix in the same way as the previous studies (Hagio and Kouzaki, 2018): The input matrix, ***W***_*inp*_, is initialized as white noise matrix and the innervation matrix, ***Z***, is initialized so that columns of the matrix uniformly distribute on the ${\mathbb{R}}^{N_{mus}}$ hypersphere.

#### 2.2.2 Synergy Model

The synergy model shares the same structure with the non-synergy model for input and the first intermediate layer. The difference is an additional layer (synergy layer) inserted just after the M1 layer, which mimics the spinal interneurons (Figure 3C, yellow-colored neurons). The cortical input matrix to the synergy layer, ***Z*****_*syn*_**, was defined similarly to the non-synergy model but normalized such that the average force amplitude of M1 neurons is identical to that of non-synergy model (Hagio and Kouzaki, 2018). This ***Z*****_*syn*_** normalization is necessary for a fair comparison of the non-synergy model and the synergy model. Furthermore, it allows us to exclude the average scale of torque, $\bar{T_{mod}}$ (Equation 8), exerted by each synergies of interest, *T*_*mod*_*i*__ (Equation 9)


(8) $$\begin{array}{l} \bar{T_{mod}}=\frac{1}{N_{mod}}\Sigma_{i=1}^{N_{mod}}|T_{{mod}_{i}}| \end{array}$$

(9) $$\begin{array}{l} T_{{mod}_{i}}=M\left(\theta \right)F_{iso}K_{AL}W_{syn_{i}} \end{array}$$

from consideration. Muscle synergies, ***W*****_*syn*_**, are provided by either non-negative matrix factorization (NMF) or a random combination of feasible muscle activation. We refer to muscle synergies extracted by NMF as “NMF-derived synergies”. On the other hand, muscle synergies comprise randomly selected feasible muscle activation as “feasible synergies” in this study (see Experiment 2). Similar to the non-synergy model, the network output is transformed into muscle activation with non-negative constraints in a non-linear network.


(10) $$\begin{array}{l} a=\left\lfloor W_{syn}Z_{syn}W_{inp}x\right\rfloor \end{array}$$

The NMF-derived synergies were extracted from the optimized non-synergy model. The non-synergy model was optimized by training 25,000 trials, and the last 100 trials for muscle activation data were used to extract muscle synergies (Hirashima and Oya, 2016; Song et al., 2022). We set the number of NMF-derived synergies and feasible synergies at four where the averaged variance accounted for (VAF), which is calculated as VAF = 1 - SSE/SST, exceeds 0.95 (Rodriguez et al., 2013; Sy et al., 2016) (Figure 3D). SSE denotes the sum of the squares of residual errors, and SST is the sum of the square differences between each data point and the overall mean. In this study, we adopted the more strict heuristic threshold, which is less frequently used, because a lenient threshold could overlook important components for task achievement (Barradas et al., 2020). In our model, lenient threshold such as *VAF* = 0.90 provides only three muscle synergies that are insufficient to achieve the two-dimensional motor task (Figure 3D).

#### 2.2.3 Training procedure

The neural network is trained for 25,000 trials, and the input weight matrix at *t*-th learning trial, ***W***_*inp*_(*t*), is updated using the error feedback-with-decay algorithm.


(11) $$\begin{array}{l} W_{inp}\left(t+1\right)=-\alpha \frac{\partial J_{e}}{\partial W_{inp}\left(t\right)}-\beta W_{inp}\left(t\right) \end{array}$$

Where α denotes learning rate and β denotes decay rate. *J*_*e*_ denotes the error cost that is calculated by the error vector at *t*-th trial, ***e***(*t*) = **τ**(*t*) − ***T***(*t*), between output torque and input torque, $J_{e}=1/2e{\left(t\right)}^{⊤}e\left(t\right)$. The input matrix is updated in accordance with the gradient of the error cost (The first term of Equation 11).

The second term is a weight decay term that minimizes motor effort (Hirashima and Nozaki, 2012). Learning rate and decay rate were set to α = 20 and β = 1.0 × 10^−4^, respectively.

This research defines motor error as the norm of the error vector. The learning speed of the network, *v*, is determined by calculating the exponential coefficient of an approximation function that represents the change in learning error across trials, |*e*_*t*_| (Equation 12) (Takiyama and Okada, 2012).


(12) $$\begin{array}{l} |e_{t}|=\gamma_{1}exp\left(-vt\right)+\gamma_{2} \end{array}$$

### 2.3 Direction bias and power bias

To assess how the mechanical properties of the modules affect the performance of the network, we proposed two criteria that quantify the mechanical properties of the modules in the task space.

#### 2.3.1 Direction bias

**Figure 4:**
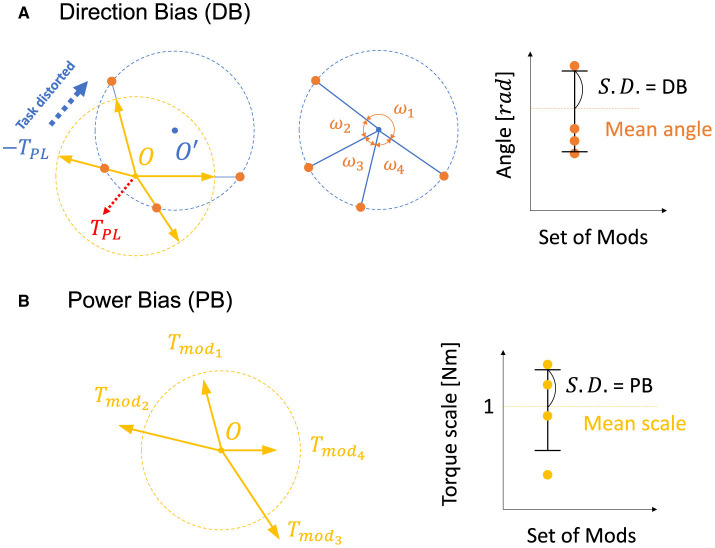
Visual description of direction bias and power bias. **(A, B)** Description of direction bias. The yellow dotted circle represents the circle of the torque production target with a radius of 1[Nm] (Figure 3A), and the yellow arrows represent the torque direction of modules, ***T***_*mod*_. The blue dotted circle represents a circle of torque that the network actually generates to compensate for the passive force (red dotted arrow, ***T***_*PL*_) and to realize the desired torque targets. Orange dots represent intersections of the blue dotted circle and the vectors of modules, ***T***_*mod*_.

The previous research (Hagio and Kouzaki, 2018) used a non-linear network and partially demonstrated that a more effective set of modules for motor learning is associated with the angular regularity of forces of a set of modules in the task space. These findings are true for a simplified musculoskeletal model and partially true even for our musculoskeletal model. Inspired from their work and considering the mathematics of error-based learning of a linear neural network, we first conceived one novel criteria named “Direction Bias (DB)”. The basic concept of DB is illustrated in Figure 1 at the bottom. DB quantifies how the set of module torques in use is irregular in direction in the task space. If the forces of the modules are sparsely distributed in the task space (Figure 1, left one at the bottom), DB evaluates the given module set as the better one, while the biased one (Figure 1, right one at the bottom) is considered worse. However, we heuristically found the complexities of the realistic musculoskeletal system, i.e., the contribution of passive force (Equation 2), affect how the forces of modules should be distributed in the task space because of the non-negative constraint of the muscle activation (Equations 6, 10). Qualitatively, this non-negative constraint rectify the muscle control signals, and it forces the network to compensate for the passive force. For example, if the desired torque targets, τ, distribute on a circle's circumference like Figure 3A, the network has to generate **τ−*T*_*PL*_** to realize the desired torque. In the end, the torques produced by the network will be placed on another circle's circumference such that the task circle is shifted toward **τ−*T*_*PL*_** (Figure 3A, blue-dotted circle). Therefore, we hypothesized that the set of synergies whose torque vectors regularly cover this shifted circle will be efficient for achieving the task. Direction bias (DB) formulates this idea, and it evaluates how the torques of modules, ***T***_*mod*_, *do not* regularly distribute on the torque circle where output torque of the network eventually need to be placed for producing the desired torques on the circumrefence. The calculation of DB is done as follows: First, we calculate the intersection points of torque vector of modules, ***T***_*mod*_, and the shifted circle, |τ−***T***_*PL*_| = 1[*Nm*] (Figure 4A left). Next, we calculate the angles of neighboring intersection points, ω_*i*_, on the circle's circumference (Figure 4A middle). Finally, we calculate DB as the standard deviation of the angles of neighboring points on the circle's circumference (Figure 4A right) (Equation 13). Note that it provides an identical indication to the bias in mechanical direction of modules (Hagio and Kouzaki, 2018) when the passive force is zero:


(13) $$\begin{array}{l} DB=\frac{1}{N_{mod}}\sqrt{\overset{N_{mod}}{\underset{i=1}{\sum }}{(\omega_{i}-\bar{\omega })}^{2}} \end{array}$$

where $\bar{\omega }$ denotes mean of ω.

#### 2.3.2 Power bias

DB does not take care of the scale of the module forces. However, from the linear neural network theory, the scale of the module torque also determines the characteristics of the network (e.g., eigenvalues of the system matrix) in the same way as the direction of the forces, which DB assesses. Therefore, we extended the idea of mechanical regularity of the modules from the angular perspective to the gain perspective. The concept is illustrated in Figure 1. In general, if the gain of the modules is not even (Figure 1 right), the gains are possibly worse for the task. To investigate how the unevenness of the forces of the modules affect motor performance, we proposed Power Bias (PB) to evaluate how the gains of the forces of the modules “are not” regular. PB is computed as the standard deviation of the gains of the modules (Figure 4) (Equation 14):


(14) $$\begin{array}{l} PB=\frac{1}{N_{mod}}\sqrt{\overset{N_{mod}}{\underset{i=1}{\sum }}|T_{mod_{i}}-\bar{T_{mod}}{|}^{2}} \end{array}$$

Where *T*_*mod*_*i*__ denotes the torque of the *i*-th modules and $\bar{T_{mod}}$ denotes the average torque of the modules.

### 2.4 Experimental procedure

#### 2.4.1 Experiment 1: synergy model vs. non-synergy model

To investigate the performance of the modularity in our musculoskeletal system, we conducted a standard comparative experiment. The non-synergy model was trained first (i.e., Section 2.2.3) at the Base Posture (BP). Next, we extracted NMF-derived synergies from this non-synergy model. We prepared one synergy model, which learns how to control muscle synergies optimized for BP, and we provided it with extracted synergies. This model is referred to as “BP mods” in this study. Regarding the measurement of DB and PB of non-synergy model, we calculated PB and DB of the synergy model whose ***W***_*syn*_ is *N*_*mus*_ dimensional identity matrix, $I\in {\mathbb{R}}^{N_{mus}\times N_{mus}}$ instead.

Furthermore, to discuss why muscle synergies extracted from specific biomechanical contexts sometimes fail to perform well in other contexts from the mechanical viewpoint, we prepared two additional synergy models and two sets of NMF-derived synergies for the models. These additional sets of synergies are extracted from two non-synergy models optimized for two postures different from BP. We provided synergy model with these two module sets to the motor learning task at the BP. At one of the postures, we refer to as “Fully Extended (FE, Figure 2 middle)”,θ_*FE*_, the elbow is horizontally extended 90 degrees from the BP. At the other posture, we refer to “Mildly Extended (ME, Figure 2 right)”,θ_*ME*_, the elbow was extended 45 degrees from the BP. These models are referred to as “FE mods” and “ME mods”, respectively, in this study. The three synergy models, which respectively control muscle synergies extracted from a non-synergy model optimized for FE, ME, or BP, were trained at the BP and iterated 30 times. We compared the learning performance across these three synergy models and the non-synergy model, and we also measured PB and DB of the utilized synergies of the models and the non-synergy model for analysis. Note that the synergy model learns only how to control the provided muscle synergies through the optimization of ***W***_*inp*_, and the synergies are not updated during the learning. Therefore, the difference in learning performance across the synergy models depends on their synergies. Comparison of the learning performance of across models were conducted with analysis of variance (ANOVA) and post hoc test. On the other hand, the performance of the synergy network and the non-synergy network was analyzed with the Wilcoxon signed rank test.

#### 2.4.2 Experiment 2: correlation of performance of modularity with PB and DB

To demonstrate to what extent PB and DB explain the performance of non-linear networks in a realistic musculoskeletal system, we prepared multiple sets of feasible synergies and conducted learning simulations of the synergy model.

The feasible synergies can be categorized into two groups: One is the synergies, which we refer to as “FSforDB”, and it is used for verification of DB. It consists of *N*_*mod*_ synergies whose amplitude of exerting torques remain consistent while their directions vary. In other words, among the different FSforDB, PB are preserved and only DB vary.

**Figure 5:**
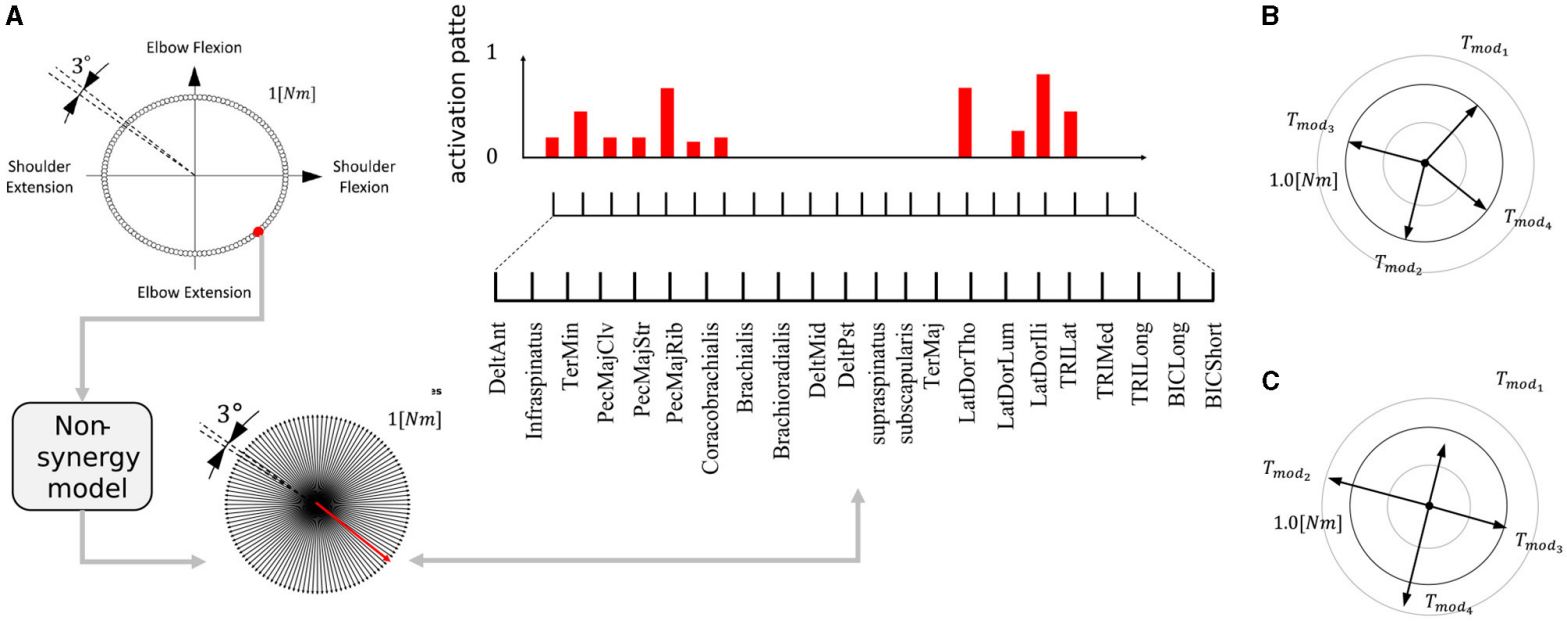
Making of the feasible synergy set. **(A)** Making muscle activation pattern dataset. The red dot, arrow, and bars are example of making one activation-torque data. Each corresponds to motor target the non-synergy network receives, the torque produced by the network, and the muscle activation for producing the motor target, respectively. This procedure is iterated for all the motor targets (upper left), and the non-synergy model trained to produce the given torque computes necessary motor activation pattern (upper right). **(B)** Example of a set of four feasible synergies used for verification of DB. *N*_*mod*_ muscle activation patterns are randomly selected from the normalized muscle activation data is so that the amplitude of torque of each synergy is 1 [Nm]; ***T***_*mod*_*i*__ = 1. **(C)** Example of four feasible synergies used for verification of PB. The muscle activation patterns are selected to satisfy *DB* = 0 and scaled randomly so that mean amplitude of torque is 1 [Nm]; $\bar{T_{mod}}=1$.

Generation of this type of synergies (FSforDB) is as follows. First, we generated a dataset of muscle activity patterns necessary to exert force in any direction. Specifically, torque targets were placed at intervals of 3° along the circumference of a torque circle with a radius of 1 [Nm] (Figure 5A, upper left). By training a non-synergy model to produce each target torque, we made the dataset of 120 muscle activity patterns required to exert force in each direction. After that, we randomly selected *N*_*mod*_ muscle activation patterns from the dataset. The example of FSforDB is illustrated in Figure 5B. If the synergy model sufficiently learn the task of Figure 3A using the set of selected muscle activation patterns, we admitted it as the valid set of FSforDB for verification of DB. In this research, we determined the sufficiency of the learning performance of the synergy network by using a threshold, which we refer to as the “feasibility cut-off.” In other words, if the synergy network, utilizing a set of selected muscle activation patterns, fails to learn the task so that the average torque error remains below the value for feasibility cutoff, the selected muscle activation patterns are excluded from the dataset of the feasible synergies. We established the feasibility cutoff value as 0.1 [Nm] and applied it to the generation of FSforDB. This sorting is necessary for analysis because an ineffective synergy set can cause learning of the synergy network to easily drop into bad local optima, where the learning was not sufficiently progressed.

The other type of feasible synergies set, which we refer to as “FSforPB”, is used for verification of PB. It consists of *N*_*mod*_ synergies whose amplitude of exerting torques vary while their directions remain consistent. In other words, among the different FSforPB, DB are preserved and only PB vary, in contrast with FSforDB. Generation of this type of synergies (FSforPB) is as follows. We first selected *N*_*mod*_ activation patterns from the dataset of 120 muscle activation patterns, which we made for generation of FSforDB, so that *DB* = 0. Next, we randomly scaled their gains within the interval of [0.5, 1.5] so that the average torque gain of the synergies, $\bar{T_{mod}}=1$, becomes 1 [Nm]. The example of FSforPB is illustrated in Figure 5C. Finally, similar to generation FSforDB, if the synergy network can sufficiently learn the task of Figure 3A using the generated synergies set, we admitted it as the valid set of FSforPB for verification of PB. As for the feasibility cutoff for FSforPB, in common with FSforDB, we set the threshold value as 0.1 [Nm].

To verify our criteria, we trained the synergy model 30 times for each set of synergies of either sets of FSforPB or sets of FSforDB. In this reserach, We generated 30 sets of FSforPB and we generated 90 sets of FSforDB. The learning performance of each synergy model is represented as the median of 30 times training. We analyzed the correlation of our criteria with the learning performance of the synergy models. The statistical analysis is performed using multiple regression analysis with F-test.

#### 2.4.3 Experiment 3: effect of dimensionality of modules on PB and DB

To investigate whether the explanation capability of PB and DB are robust to the number of modules, we conducted motor learning simulations while changing the number of modules, DB and PB. We iterated each simulation 30 times and analyzed the data with multivariate analysis. We investigated the correlation when *N*_*mod*_ = 8, 15, 22. Note that the synergy model that controls the same number of synergies with the muscles, *N*_*mod*_ = 22, is not equivalent to the non-synergy model unless the synergy matrix, ***W***_*syn*_, is the identity matrix. The number of samples is the same as in experiment 2 (30 samples for PB and 90 samples for DB). We calculate the *p*-value with *F*-test and *R*^2^ for each number of modules.

In addition, to test whether the dimensionality of the synergy network affects the accuracy and learning speed of the synergy model, we compared the performance of the synergy network when both PB and DB were zero at each number of modules. In other words, we compared the performance of the synergy network when both PB and DB were zero at each number of modules. We analyzed the data with analysis of variance (ANOVA) and post hoc test.

#### 2.4.4 Experiment 4: independence test of DB and PB

To investigate whether PB and DB independently affect the learning performance, we trained the synergy network with module sets that provide various values of PB and DB. We conducted this investigation for the case of *N*_*mod*_ = 4, 8, 15, and 22. To generate new feasible synergy sets whose DB and PB vary across each synergy set, we randomly sampled 60 FSforDB without replacement and changing PB of them within the interval of (0.5 1.5). The feasibility cutoff value for this new synergy was set as 0.15 [Nm]. We conducted the learning simulation of the synergy model 30 times, and we analyzed the median data with multiple regression analysis. The data of the error or the learning speed, here we denote ***X***, is regressed by a linear function of PB and DB:


(15) $$\begin{array}{l} X=k_{1}PB+k_{2}DB+k_{3} \end{array}$$

Where *k*_*i*_ denotes regression coefficients. We used the F-test to calculate the p-value of the regression. Furthermore, we also calculated the Variance Inflation Factor (VIF) of PB and DB to test the multicollinearity.

#### 2.4.5 Experiment 5: effect of the scale of the passive force element on PB and DB

To demonstrate the effect of the scale of the passive force on the explanation capability, we changed the scale of the passive force element so that the passive force derived torque, ***T*****_*PL*_** = 0.3 and 0.7 [Nm] (Equation 5). The number of modules, *N*_*mod*_, is determined as four. We provide the synergy model with the same modules as the set used in section 2.4.2: 90 samples for DB and 30 modules for PB, and examined how the correlations in learning performance and the criteria changes with the scale of ***T*****_*PL*_**.

## 3 Results

### 3.1 Musculoskeletal model with passive force well reproduces isometric mechanics of OpenSim

**Figure 6:**
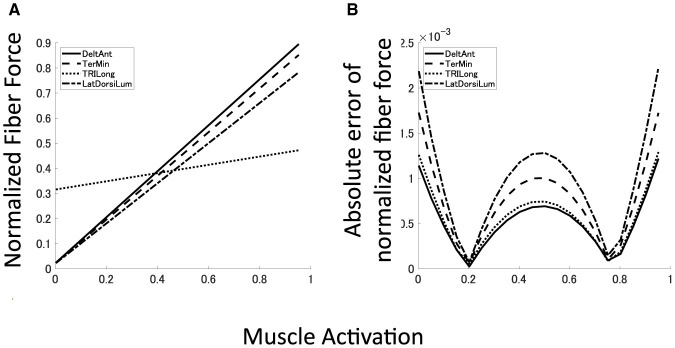
Approximated muscle mechanics and regression accuracy. **(A, B)** Four examples of approximated muscle mechanics and its absolute error of normalized fiber force. Solid line represents DeltAnt, long dashed line for TerMin, dotted line for TRIlong, and chain line for LatDorsiLum.

To demonstrate the reproduction capability of our plant for the musculoskeletal model, the mean error and mean absolute deviation (MAD) of each muscle force at the base posture were calculated. Furthermore, *R*^2^ value and *p*-value of linear hypothesis test based on F-test (Table 1). All the muscles are well reproduced by linear regression with high reliability (*R*^2^ > 0.99, *p* < 0.05). Although most of the large approximation errors of the normalized muscle force concentrate around *u* = 0, 0.5, 1.0, its approximation error keeps within 2.5 × 10^−3^ (Figure 6B), and mean error of normalized muscle forces keep within 5% and MADs keeps within 6%. These results indicate that our musculoskeletal model reproduces characteristics of the musculoskeletal model, including a significant property, i.e., the passive force (Figure 6A) in the isometric force production.

**Table 1:** Approximation accuracy of the musculoskeletal model.

**Muscle name**	**Mean error [*%*]**	**MAD [*%*]**	***R* ^2^**	***p*-value**
DeltAnt	0.4083	0.5289	1.0000	4.3059 × 10^−51^
DeltMid	4.0300	5.4351	0.9994	4.0909 × 10^−32^
DeltPst	0.1557	0.2032	1.0000	2.9574 × 10^−59^
supraspinatus	2.1961	2.8877	0.9998	4.3559 × 10^−37^
infraspinatus	0.2593	0.3389	1.0000	4.7510 × 10^−55^
Subscapularis	0.2108	0.2759	1.0000	9.2134 × 10^−57^
TerMin	0.6371	0.8315	1.0000	1.5181 × 10^−47^
TerMaj	0.0158	0.020	1.0000	4.4394 × 10^−78^
PecMajClv	0.0121	0.0158	1.0000	2.1650 × 10^−80^
PecMajStr	0.4289	0.5603	1.0000	7.6528 × 10^−51^
PecMajRib	0.5998	0.7855	1.0000	4.6422 × 10^−48^
LatDorsiTho	0.4225	0.5537	1.0000	5.9591 × 10^−51^
LatDorsiLum	0.8733	1.1382	1.0000	7.8627 × 10^−45^
LatDorsiIli	0.3257	0.4263	1.0000	3.5705 × 10^−53^
Corabrachialis	0.2042	0.2786	1.0000	7.3990 × 10^−58^
TRILong	0.1418	0.0692	1.0000	2.8085 × 10^−36^
TRILat	0.4837	0.6313	1.0000	6.8502 × 10^−50^
TRIMed	0.3710	0.4867	1.0000	3.5051 × 10^−52^
BICLong	0.9971	1.2906	1.0000	1.4896 × 10^−43^
BICShort	2.5013	3.3371	0.9998	7.1196 × 10^−37^
Brachialis	0.3893	0.5053	1.0000	6.4226 × 10^−43^
Brachioradialis	1.0909	1.4146	1.0000	6.4226 × 10^−43^

### 3.2 Performance of modularity depends on mechanical properties of muscle synergies—Experiment 1

**Figure 7:**
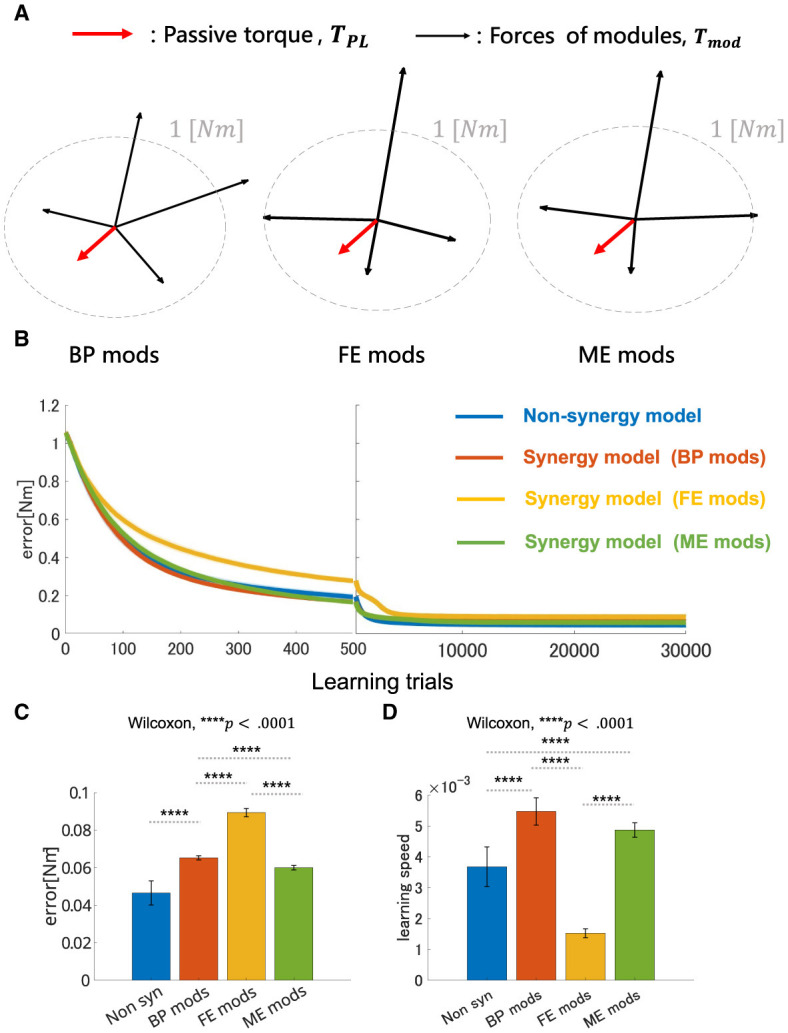
Performance comparison of non-synergy network and synergy network with different module sets in motor learning simulation with non-linear neural networks. **(A**) Visualization of torque of module sets. Red arrows represent the vector of the passive torque, ***T***_*PL*_, at BP. Black arrows represent the vectors of the torque which synergies of the models, i.e., BP mods, FE mods, and ME mods, exert, ***T***_*mod*_. Dashed gray circle represents the circle where desired torque targets, **τ**, are placed along whose radius is 1 [Nm]. **(B)** Plot of trial-dependent change of error. The blue solid line represents a mean trial-dependent loss of the non-synergy network, the orange solid line for the synergy network with base posture modules (BP mods), the yellow solid line for the synergy network with fully extended modules (FE mods), and the green solid line for the synergy network with mildly extended modules (ME mods). Shade regions represent the standard deviation of the trial-dependent loss change. **(C, D)** Comparison of non-synergy network and the synergy networks with different modules in final error and learning speed, respectively.

The overall performance of the non-synergy model and synergy models with three NMF-derived synergies are shown in Figure 7, and the mechanical properties of each module and the performances were summarized in Figure 7D and DB and PB of those sets of modules and individual muscles are summarized in Table 2. Across the three synergy models, significant differences in performance in motor achievement and learning speed are found (Figure 7B; for motor achievement, *p* = 1.70 × 10^−80^, for learning speed *p* = 4.39 × 10^−68^, ANOVA). The synergy model with NMF-derived synergies at the base posture (BP mods) significantly degrades motor achievement compared with the non-synergy model (*p* = 3.02 × 10^−11^, Wilcoxon). The synergy model with NMF-derived synergies of the elbow fully extended posture (FE mods) showed the worst motor achievement across the three module sets. Similarly, although the synergy model with the NMF-derived synergy of elbow mildly extended posture (ME mods) provides the best across the three modules, it significantly degraded the motor accuracy from the non-synergy model (*p* = 3.39 × 10^−6^, Wilcoxon).

**Table 2:** Power bias and direction bias of modules and non-synergy model.

**Network type**	**Direction bias**	**Power bias**	**Mean error [Nm]**	**Learning speed**
Non-synergy	16.3636	0.9904	0.0465	0.0037
BP mods	6.0308	0.3387	0.0653	0.0055
ME mods	26.6063	0.4386	0.0600	0.0049
FE mods	42.1959	0.4901	0.0894	0.0015

On the other hand, the BP mods significantly promoted the learning speed compared with the non-synergy model (*p* = 4.98 × 10^−11^, Wilcoxon). The synergy model with MD modules also promoted the motor learning (*p* = 5.07 × 10^−10^, Wilcoxon). However, the synergy model with FE modules significantly slowed down the motor learning compared with individual control (*p* = 3.02 × 10^−11^, Wilcoxon). The results indicate that modularity may degrade motor achievement, and its learning promotion depends on the synergies used in a complex musculoskeletal model. Furthermore, the performance of modularity largely depends on the mechanical properties of the muscle synergies.

### 3.3 Performance of modularity correlates with DB and PB—Experiment2

**Figure 8:**
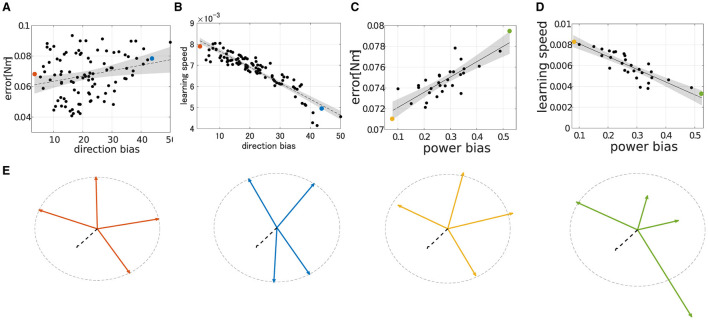
Scatter plot of performance in learning simulation of synergy network when *N*_*mod*_ = 4. **(A, B)** Correlation of final error or learning speed and direction bias. **(C, D)** Correlation of final error or learning speed and power bias. The dashed line and shade interpolated in each figure represent first-order regression and 95% confidence intervals of the scatter plot. **(E)** Amplitude and direction of the synergies of interest. The black dotted arrow represents the passive torque, ***T***_*PL*_. The orange, cyan, yellow, and green arrows represent synergies dotted in **A–D**, respectively.

To qualitatively demonstrate how the performance of modularity correlates with DB and PB, we conducted extensive simulations of modularity with four modules at the base posture while changing their DB or PB. The scatter plot of the median of the learning performance of modularity and the criteria, i.e., DB and PB, was summarized in Figure 8. While DB showed a strong negative correlation with learning speed (Figure 8C; *R*^2^ = 0.845, *p* = 6.97 × 10^−40^, *N* = 90, F test), it shows an almost no correlation with motor achievement (Figure 8A *R*^2^ = 0.0635, *p* = 1.13 × 10^2^, *N* = 90, F test). In contrast, PB consistently and strongly correlated with both motor achievement and learning speed (for motor achievement *R*^2^ = 0.7608, *p* = 4.32 × 10^−3^ and for learning speed *R*^2^ = 0.7471, *p* = 1.05 × 10^−7^, *N* = 30, F test). These results indicate that PB and DB are effective criteria for evaluating the modules and predicting the performance of modularity with four modules at the base posture. Specifically, while motor achievement mainly depends only on PB, learning speed depends on both PB and DB.

### 3.4 Impacts of dimensionality on the performance, DB and PB are consistent—Experiment 3

**Figure 9:**
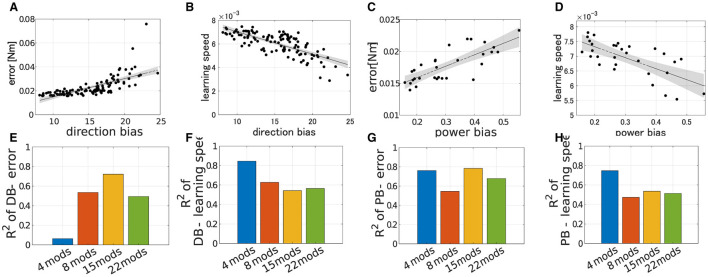
Correlation of learning performances and DB or PB with different numbers of modules. **(A–D)** Correlation of final error or learning speed and criteria when *N*_*mod*_ = 22. **(E–H)** Bar plot of *R*^2^ error or learning speed with different scale of the passive force (blue; *N*_*mod*_ = 4, orange; *N*_*mod*_ = 8, yellow; *N*_*mod*_ = 15 and green; *N*_*mod*_ = 22).

We verified the robustness of the explanation capabilities of the criteria to the number of modules. The results are summarized in the Figure 9. Figures 9A–D illustrates the correlation between the performance of the modularity and PB or DB when *N*_*mod*_ = 22. Similar to the case of *N*_*mod*_ = 4, learning speed strongly correlates with both DB and PB (Figures 9C, D; for DB *R*^2^ = 0.5647, *p* = 9.98 × 10^−19^ and for PB *R*^2^ = 0.5133, *p* = 8.46 × 10^−6^, F test), and we also found strong correlation of PB and motor achievement (Figure 9A). However, in contrast, DB correlates with motor achievement (Figure 9A). In other cases with different modules (*N*_*mod*_ = 8 and 15), we also found similar tendencies (Figures 9E, H) except for the correlation of DB and motor achievement *N*_*mod*_ = 4 (Figure 9E).

**Figure 10:**
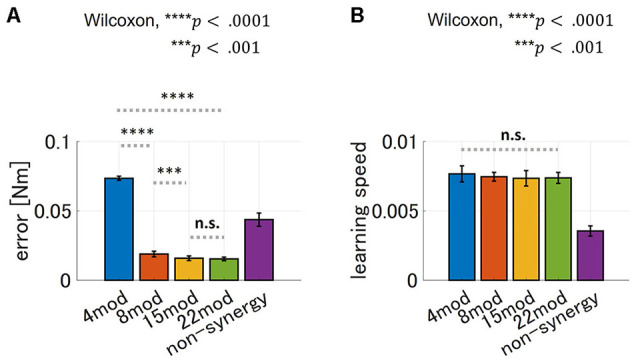
Comparison of performance of synergy network with different number of DB-PB minimizing modules. **(A, B)** Bar plot of final error and learning speed of synergy network using different number of DB and PB minimizing module sets, respectively (blue; *N*_*mod*_ = 4, orange; *N*_*mod*_ = 8, yellow; *N*_*mod*_ = 15 and green; *N*_*mod*_ = 22). For comparison, we added the performance of non-synergy model to the graph as the purple bar plot.

Furthermore, we tested the performance of modularity with different numbers of modules with zero PB and DB. The result is summarized in Figure 10. As for motor achievement, we found significant difference of motor achievement performance across groups (*p* = 4.66 × 10^−^18, ANOVA), and varies across the case of *N*_*mod*_ = 4, 8 and 15 (Figure 10A; comparison of *N*_*mod*_ = 4 and *N*_*mod*_ = 8, *p* = 3.39 × 10 − 6, and comparison of *N*_*mod*_ = 8 and *N*_*mod*_ = 15, *p* = 4.94 × 10 − 4, Wilcoxon). Motor achievement performance of *N*_*mod*_ = 15 and *N*_*mod*_ = 22 did not show significant difference (*p* = 5.61 × 10^−1^, Wilcoxon). On the other hand, as for learning speed, there is no significant difference across the groups (*p* = 4.45 × 10^−1^, ANOVA). These results suggest that maximum learning speed does not depend on the number of modules, while motor achievement does.

### 3.5 Independent impact of DB and PB on performance of modularity—Experiment 4

**Figure 11:**
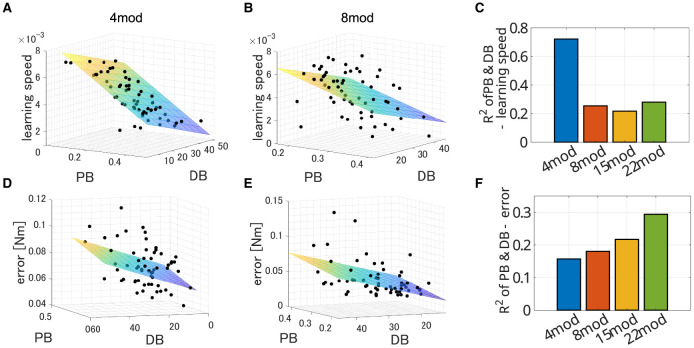
Estimated performance of synergy network with module sets of various PB and DB when *N*_*mod*_ = 4, 8, 15, and 22. **(A, B)** Three-dimensional scatter of learning speed of synergy network when *N*_*mod*_ = 4 and 8, respectively. The colored surface represents a two-dimensional plane estimating the performances of the networks.**(D, E)** Three-dimensional scatter of learning error of synergy network when *N*_*mod*_ = 4 and 8, respectively. The colored surface represents a two-dimensional plane estimating the performances of the networks. **(C, F)** Comparison of explanatory power of the model among the synergy model with different number of modules.

In all cases, PB and DB are independent and there is no multicollinearity (VIF = 1.0008 for *N*_*mod*_ = 4, 1.0005 for *N*_*mod*_ = 8, 1.0677 for *N*_*mod*_ = 15 and 1.0436 for *N*_*mod*_ = 22). Figure 11 illustrates example of the spatial distribution of the performance of modularity in the PB-DB space and comparison of explanation power among the different number of modules. The planes in each figure represent the approximated 3D planes of predicted performances of modularity against PB and DB that assume PB and DB independently affect the performance of modularity. While the independent description of motor achievement by PB and DB was not so successful (Figure 11B; *R*^2^ = 0.15699, *p* = 7.7 × 10^−3^. F test, *N* = 60), it successfully explained the major variance of learning speed (Figure 11A; *R*^2^ = 0.7220, *p* = 1.43 × 10^−16^, F test, *N* = 60). On the other hand, the explanation power of criteria decreased when the synergy model controls more modules (Figures 11C, F). The independent model of PB and DB exhibit a limited explanation power for a limited part of the variance of both learning speed (*R*^2^ = 0.254, *p* = 2.37 × 10^−4^ for *N*_*mod*_ = 8, *R*^2^ = 0.217, *p* = 9.44 × 10^−4^ for *N*_*mod*_ = 15, and *R*^2^ = 0.280, *p* = 8.60 × 10^−5^ for *N*_*mod*_ = 22) and learning error (*R*^2^ = 0.1803, *p* = 3.46 × 10^−3^ for *N*_*mod*_ = 8, *R*^2^ = 0.2169, *p* = 9.41 × 10^−4^ for *N*_*mod*_ = 15, and *R*^2^ = 0.294, *p* = 4.93 × 10^−5^ for *N*_*mod*_ = 22).

### 3.6 Scale of the passive force affects the effectiveness of the criteria—Experiment 5

**Figure 12:**
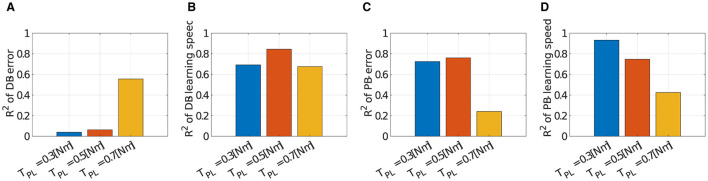
Correlation of learning performances and DB or PB with different scale of the passive force when *N*_*mod*_ = 4. **(A–D)** Bar plot of *R*^2^ value of error or learning speed with different number of modules (blue; ***T*****_*PL*_** = 0.3[*Nm*], orange; ***T*****_*PL*_ = 0.5[*Nm*]**, and yellow; ***T*****_*PL*_** = 0.7[*Nm*]). Note that ***T*****_*PL*_ = 0.5[*Nm*]** is the data identical to Figure 8.

We verified the robustness of the criteria to the scale of the passive force. Figure 12 illustrates how the explanation capabilities of the criteria change with the scale of the passive force. DB successfully explained the major variance of the learning speed data (Figure 12A; *R*^2^ = 0.6931 for ***T*****_*PL*_** = 0.3[*Nm*], *R*^2^ = 0.6768 for ***T*****_*PL*_** = 0.7[*Nm*]). However, it failed to explain the data on motor achievement except for ***T*****_*PL*_ = 0.7** [Nm] (Figure 12B; *R*^2^ = 0.0392 for ***T*****_*PL*_** = 0.3[*Nm*], *R*^2^ = 0.5557 for ***T*****_*PL*_** = 0.7[*Nm*]). On the other hand, PB relatively succeeded in explaining the variance of the data. For motor achievement, PB explained a large part of the data when ***T*****_*PL*_** = 0.3[*Nm*] (Figure 12C; *R*^2^ = 0.7252), while it only explained some parts of the data when ***T*****_*PL*_** = 0.7[*Nm*] (*R*^2^ = 0.2411). For learning speed (Figure 12D), similar to motor achievement, the explanation capability of the criteria is strong when ***T*****_*PL*_** = 0.3[*Nm*] (*R*^2^ = 0.9330), while it gets weak when ***T*****_*PL*_** = 0.7[*Nm*] (*R*^2^ = 0.4254).

## 4 Discussion

### 4.1 Passive force element of the muscle influences the selection of the module

One of the key contributions of our research is the consideration of the passive force element in muscle mechanics within the criteria and our musculoskeletal model. This element is a significant component of typical Hill-type musculoskeletal mechanics, although it has been underappreciated and neglected in some previous literature (Rokni et al., 2007; Hirashima and Nozaki, 2012; Hirashima and Oya, 2016; Hagio and Kouzaki, 2018; Song et al., 2022). In a static condition, specifically isometric torque production, the upper limb musculoskeletal mechanics (Saul et al., 2015) is simplified into an affine-like system for muscle activation. Despite this simplification, its reproducibility is high (Table 1) due to the negligible second-order or higher-order behavior in this context. As shown in Figure 6, some muscles produce non-zero force even when no control signals are provided (***a*** = 0), and these forces act as constant passive joint torque based on their joint moment arms depending on the posture of the musculoskeletal model.

The passive characteristics of muscles play a crucial role in controlling musculoskeletal mechanics by influencing impedance modification in musculoskeletal systems (Winters et al., 1988) or storing elastic energy (Roberts, 2016). Although it has recently gained renewed attention for its significant role in modeling musculoskeletal mechanics (Herbert and Gandevia, 2019; Herzog, 2019) and is gradually being considered in the context of computational control of realistic musculoskeletal models (Al Borno et al., 2020; Fischer et al., 2021), how it interacts with musculoskeletal control has not been well elucidated.

However, except for a weak correlation case found in lower-dimensional modularity (i.e., *N*_*mod*_ = 4; Figures 8A, 9A), certain correlations between DB and learning error or the speed of the network directly demonstrate the necessity of efficiently occupying task space considering elastic components by utilized modules for higher control performance. This tendency is preserved across different scales of passive-derived torque for learning speed (Figure 12B), while it fails to explain the data variance for learning accuracy (Figure 12A). This failure may stem from the limitation of the network's dimensionality because the explanatory capability of DB for learning error is extremely low only when *N*_*mod*_ = 4, compared with higher-dimensional modularity (Figure 9A). It is considered that this limitation does not cast doubt on the effectiveness of DB.

The other criterion we proposed, i.e., PB, which quantifies the evenness in torque amplitude of the utilized modules, generally succeeds in explaining the data tendencies of learning performances. In contrast to DB, its validity is relatively preserved across the number of network dimensionalities (Figures 9C, D). However, it tends to decline as the amplitude of the passive force increases (Figures 12C, D). This is possible because the definition of PB disregards the passive force element of the muscles, as the results show. Overall, the passive element of muscle significantly affects the selection of the direction of the module torque for the improvement of learning performance, whereas it does not strongly affect the selection of the amplitude of the module torque, at least in the region where the action of passive force is weak. It seems that the direction regularity and power evenness of the torque of the modules serve as a good guideline for modularity in the given task setting.

### 4.2 Qualitative description of DB and PB

The previous study (Hagio and Kouzaki, 2018) suggested that the more sparsely the modules exert the forces in the task space, the more accurate and faster motor learning the network obtains. This is the basis of the concept of DB, and it derives from learning characteristics of a neural network. For instance, in a shallow linear neural network, the forces of the modules, ***F*****_*iso*_*K*_*AL*_*W*_*syn*_**, largely affect the eigenvalues of the system matrix of the network, ***M*****(θ)*F*_*iso*_*K*_*AL*_***W*_*syn*_*Z*_*syn*_*W*_*inp*_, which dominantly determines the learning speed and largely affects the accuracy of the network. If the forces of the modules are biased in a specific direction, the motor targets in the module-concentrated direction should be achieved more accurately and faster than the other quadrants because the eigenvalue of the system matrix corresponding to the module-concentrated gets high while the other gets low under the ***Z*****_*syn*_** normalization.

**Figure 13:**
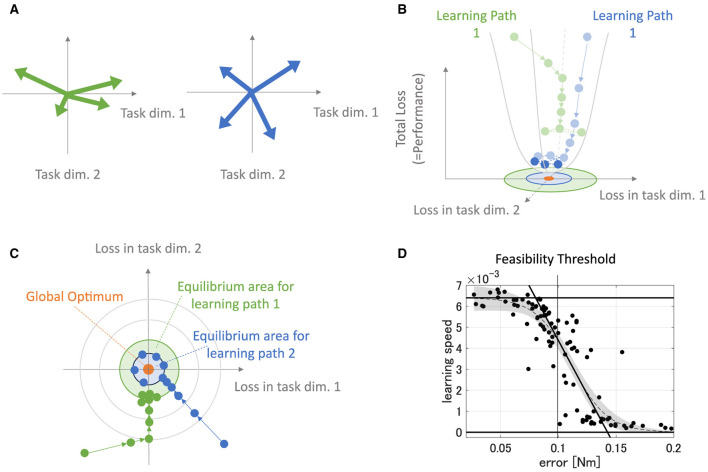
Description of learning dynamics unique to modules. **(A)** Sample of the module sets. The green-colored set of modules has high PB and DB, while the blue-colored set of modules has relatively lower PB and DB. **(B, C)** Illustration of path-dependent learning performances in 3D-shapes **(B)** and 2D-shapes **(C)**. The green path represents the learning path of the network that is provided with the biased modules [**(A)**, green arrows], and the blue path represents the learning path of the network that is provided with not-biased modules [**(A)**, blue arrows]. **(D)** The error and the learning speed relationship in the non-linear neural network that is not strongly affected by the passive force of the plant.

This description is illustrated in Figure 13. In this case, the module set is biased toward the direction of a normal basis (Figure 13A, left). This module set provides a large eigenvalue in the direction while it provides a small eigenvalue in the vertical direction. These biased eigenvalues provide the learning path with fast learning in the force-concentrated direction and slow learning in the vertical direction (Figures 13B, C, green arrows), requiring many steps for overall convergence. On the other hand, the module set unbiased in a specific direction (Figure 13A, right) provides even eigenvalues in the whole direction in the task space. These even eigenvalues provide the learning path with fast convergence for all directions, requiring fewer steps for overall convergence (Figures 13B, C, blue arrows). Importantly, the more learning proceeds, the less update the network has, resulting in a learning equilibrium that derives from forgetting and learning (Figures 13B, C, shaded circle). Weight update of the biased modules soon gets equilibrium with the forgetting compared with unbiased modules because its effective update is smaller. Therefore, biased modules provide less accurate motor achievement and slower learning speed. Such a learning speed-motor achievement relationship is preserved even in a non-linear neural network where the passive force contribution is zero or weak enough (Figure 13D), also seen in the previous study (Hagio and Kouzaki, 2018). In the same context, PB also affects the eigenvalue distribution of the system matrix of the network. Even if the torque of the modules regularly distribute on the task space in direction, unless their gains are not even, the eigenvalues are biased and affect the performance of the modularity. This system characteristic description for learning performance is very similar to and experimentally supported by a very recent work (Barradas et al., 2024). Therefore, DB and PB can be said to have the same mathematical origin as parameters affecting eigenvalues of the system matrix.

However, we should note that the above learning speed-motor achievement description is true only to linear networks or non-linear neural networks where the passive force is zero, negligible, or compensated. If the plant is non-linear, and the passive force is significant, its plausibility can be limited due to the contributions of biomechanical complexities such as changes in moment arm, passive force, and active force coefficient or the non-linearity of the muscles. These components function computationally as an affine transformation (Bebis et al., 1999) and typical rectifying units, such as ReLU layers (Dittmer et al., 2019). These characteristics would interact to affect the learning path of the network and make it difficult to predict what kind of system property is necessary for a better control performance.

To address this question, this study extend the idea of the eigenvalues of the system matrix to the non-linear neural network by dividing the property of the system matrix into two factors; regularity in direction of torque of modules and their power evenness. Through the experiment, as discussed above, although in some cases, they failed to explain the major variance of the data possibly due to the data contamination caused by feasibility cutoff in generating feasible synergies (=0.1[Nm]), the proposed criteria actually clarified the necessary property for improving the performance. Moreover, in terms of the learning behavior of an actual network, the interaction between DB and PB is not collinear; it seems they act almost independently (Figure 11; VIF = 1.0008). Therefore, while originally inspired by linear theory and heuristics, both PB and DB can be considered as independent explanatory variables that demonstrate a certain efficacy in explaining the behavior of non-linear networks and systems. Then, can we further apply the criteria to other situation such as leaning a task that is more dynamic and have multiple optimality such as walking or more dynamic reaching task? We speculate that even in the situation of more dynamic movements, it may be possible to partly expand the discussion of PB and DB when the movement optimization is done by an gradient-based learning algorithm. For example, with a proper expansion, our metrics may be applicable in a gradient-based learning of feedforward arm reaching where an ideal trajectory is already given, and they may inform us what kind of synergies are better regarding converging the error-term. However, even in this simple case, it is still unclear whether expanded metrics are easily measurable or not, and we should investigate the point in future study. In addition, while metrics are relevant only with an error-term, it will not directly explain the other optimality such as energy consumption or cost of transport, which is often used in locomotion studies. Therefore, direct use and expansion of our metrics may have a potential limitation or technological difficulties in application to such complex optimization process. Moreover, for a physiological evidence, it is necessary to see whether the criteria actually correlates with the learning curve data of primate isometric force reaching task in future.

### 4.3 Muscle synergies contribute to motor learning?

In Experiment 1, we compared the synergy network models and non-synergy models for addressing why muscle synergies extracted from a specific biomechanical contexts sometimes fail to perform well in other contexts. Overall, the synergy model, which utilizes synergies from the non-synergy model optimized for the Base Posture, i.e., BP mods, expedited the learning at the expense of motor accuracy. On the other hand, FE and ME mods showed high DB and PB (Table 2, Figure 7), and their performance significantly degraded despite the postural changes in the experiment are relatively small. It suggests that minor difference in biomechanical contexts can easily make modularity using context-specific synergies less effective. Consistent with the results of the previous study (Kutsuzawa and Hayashibe, 2022), the modularity performance largely depends on the biomechanical context from which the synergies are extracted.

However, it is important to acknowledge the limitations in our experimental protocols that may have influenced the results. We used NMF to extract each set of synergies from the non-synergy model optimized for each BP, FE, or ME. It is worth noting that NMF does not guarantee solution uniqueness or convexity of the objective function, i.e., square error of data reproduction, and determining the number of synergies is somewhat arbitrary (Barradas et al., 2020). Therefore, the extraction of synergies using NMF can miss significant components for control, and variability of synergy organization in each extraction can provide synergies with different PB and DB. These experimental limitations may have influenced the performance of each synergy model.

Furthermore, synergies extracted from the non-synergy model are features of the control and implicitly encode prior knowledge of a particular biomechanical context in which they are extracted. This means that the performance of each synergy model can partly depend on the similarity of musculoskeletal mechanics in a position of interest. This qualitative premise on simulated muscle synergies, i.e., assuming only context-specific synergies, can be distinct from the essential purpose or function of actual muscle synergies. In any case, the limitations of experimental procedures and synergy dependency in control performance obscure the contribution of muscle synergies in control. Then, what is the plausible role of muscle synergies in motor learning?

From the perspective of optimality, our results highlight the necessity of the CNS to store specific synergies for every different context, i.e., posture, as previous studies suggested (de Rugy et al., 2013; Sharif Razavian et al., 2015). If the CNS were provided with enough modules (e.g., *N*_*mod*_ > 4) that minimize both DB and PB corresponding to the posture-dependent limb mechanics, they achieved much more accurate and faster control than individually muscle-optimized control (see Figures 7C, D, 10A, B). However, strategy of storing every context-dependent control and synergy poses the problem of having to store an unlimited number of motor programs (Loeb, 1983). One possible solution to this memory storage and optimality problem is approximating a wide range of movements through generalizable synergies and subsequent optimization through context specification of the synergies as necessary.

Similar to the good-enough control strategy (Loeb, 2021), generalizable muscle synergies can be utilized across various motor contexts with reasonably good performance. While there is room for debate on such synergies, it can be potentially relevant to the shared muscle synergies observed in various static (Ting and Macpherson, 2005; Torres-Oviedo et al., 2006; Roh et al., 2012; Leonardis et al., 2020; Pham et al., 2023) and dynamic motor tasks or contexts (d'Avella and Bizzi, 2005; Chvatal and Ting, 2013; Barroso et al., 2014). Interestingly, in the lower limb static task of the cat (Torres-Oviedo et al., 2006), the experimental study reported that the rotation of the force direction exerted by shared muscle synergies is similar to limb axis rotation angles (McKay and Ting, 2008). Sohn et al. demonstrated that spatial organization of these synergies can be formed in pursuit of generalization rather than optimization for each biomechanical context (Sohn and Ting, 2016). We can relate the formation of such generalizable synergies to the minimization of DB. Suppose spatial organization of generalizable synergies is formed to minimize the average DB across similar biomechanical contexts. In that case, the CNS can efficiently generalize them for learning arbitrary similar biomechanical contexts quickly and with good enough generalization performance on average. Such DB-minimizing generalizable synergies may be represented at the medulla or spinal cord (Giszter et al., 1993; Roh et al., 2011), and robustly preserved through the subsequent development (Yang et al., 2019).

On the other hand, the specification of synergies for the optimization may be done at the higher level of motor planning, such as cortical regions where more complicated information of dynamics is encoded as synergies (Overduin et al., 2012, 2015; Amundsen Huffmaster et al., 2017). In these areas, minimization of both DB and PB is likely associated with movement optimization. However, modulation of PB may be a separate process from DB. Our experiment demonstrated that PB and DB affected learning independently to some extent. It may suggest that modulation of DB and PB of muscle synergies do not necessarily share the exact neural mechanisms. One recent study (Yaron et al., 2020) reported that, unlike the force direction of modules, the force amplitude were adjusted in supralinear form. Moreover, several suggest that sensory feedback, essential for motor planning and online control, affects the amplitude and timing of muscle synergies control while never affecting synergies' spatial organization (Inglis et al., 1994; Stapley et al., 2002; Cheung et al., 2005). PB modulation may occur in areas related to online control planning and feedback control. Meanwhile, DB modulation may take place in areas related to offline motor planning schemes, such as the selection or combination of synergies expressed in cortical areas and the coordination of the extent to which non-synergetic control elements are intervened through a cortico-motoneuronal pathway (Isa et al., 2007) or reticulospinal and vestibulospinal pathways (Cruce, 1974).

To summarize, our research has shown that the performance of fixed muscle synergy control through learning can vary significantly depending on the synergy utilized. This highlights the need to consider the role of synergy in motor control beyond a simple comparative approach to optimality. Instead, we should focus on how to exploit or overcome the dependence of synergy on control performance, such as the pursuit of generalizability or the modulation of synergy with learning. These insights have important implications for our understanding of muscle synergies and their role in motor control.

## Data availability statement

The raw data supporting the conclusions of this article will be made available by the authors, without undue reservation.

## Author contributions

AF: Writing – original draft. KK: Writing – review & editing. DO: Writing – review & editing. MH: Writing – review & editing.

## References

[B1] Al Borno M. Hicks J. L. Delp S. L. (2020). The effects of motor modularity on performance, learning and generalizability in upper-extremity reaching: a computational analysis. J. Royal Soc. Interface 17:20200011. 10.1098/rsif.2020.0011 32486950 PMC7328389

[B2] Alessandro C. Carbajal J. P. d'Avella A. (2014). A computational analysis of motor synergies by dynamic response decomposition. Front. Comput. Neurosci. 7:191. 10.3389/fncom.2013.00191 24474915 PMC3893690

[B3] Amundsen Huffmaster S. L. Van Acker G. M. III. Luchies C. W. Cheney P. D. (2017). Muscle synergies obtained from comprehensive mapping of the primary motor cortex forelimb representation using high-frequency, long-duration icms. J. Neurophysiol. 118, 455–470. 10.1152/jn.00784.2016 28446586 PMC5506266

[B4] Barradas V. R. Koike Y. Schweighofer N. (2024). Theoretical limits on the speed of learning inverse models explain the rate of adaptation in arm reaching tasks. Neural Netw. 170, 376–389. 10.1016/j.neunet.2023.10.049 38029719

[B5] Barradas V. R. Kutch J. J. Kawase T. Koike Y. Schweighofer N. (2020). When 90% of the variance is not enough: residual emg from muscle synergy extraction influences task performance. J. Neurophysiol. 123, 2180–2190. 10.1152/jn.00472.2019 32267198 PMC7311728

[B6] Barroso F. O. Torricelli D. Moreno J. C. Taylor J. Gomez-Soriano J. Bravo-Esteban E.. (2014). Shared muscle synergies in human walking and cycling. J. Neurophysiol. 112, 1984–1998. 10.1152/jn.00220.2014 25057144

[B7] Bebis G. Georgiopoulos M. da Vitoria Lobo N. Shah M. (1999). Learning affine transformations. Pattern Recognit. 32, 1783–1799. 10.1016/S0031-3203(98)00178-2

[B8] Berg C. Caggiano V. Kumar V. (2023). Sar: Generalization of physiological agility and dexterity via synergistic action representation. arXiv [preprint]. 10.15607/RSS.2023.XIX.007

[B9] Berniker M. Jarc A. Bizzi E. Tresch M. C. (2009). Simplified and effective motor control based on muscle synergies to exploit musculoskeletal dynamics. Proc. Nat. Acad. Sci. 106, 7601–7606. 10.1073/pnas.0901512106 19380738 PMC2678607

[B10] Berret B. Delis I. Gaveau J. Jean F. (2019). Optimality and modularity in human movement: from optimal control to muscle synergies, in Biomechanics of anthropomorphic systems (Springer), 105–133.

[B11] Brock O. Valero-Cuevas F. (2016). Transferring synergies from neuroscience to robotics: Comment on “hand synergies: Integration of robotics and neuroscience for understanding the control of biological and artificial hands” by m. santello et al. Phys. Life Rev. 17, 27–32. 10.1016/j.plrev.2016.05.011 27212396 PMC5542064

[B12] Chen J. Qiao H. (2020). Muscle-synergies-based neuromuscular control for motion learning and generalization of a musculoskeletal system. IEEE Trans. Syst. Man Cybernet.: Syst. 51, 3993–4006. 10.1109/TSMC.2020.2966818

[B13] Cheung V. C. d'Avella A. Tresch M. C. Bizzi E. (2005). Central and sensory contributions to the activation and organization of muscle synergies during natural motor behaviors. J. Neurosci. 25, 6419–6434. 10.1523/JNEUROSCI.4904-04.2005 16000633 PMC6725265

[B14] Chvatal S. A. Ting L. H. (2013). Common muscle synergies for balance and walking. Front. Comput. Neurosci. 7:48. 10.3389/fncom.2013.00048 23653605 PMC3641709

[B15] Cruce W. (1974). A supraspinal monosynaptic input to hindlimb motoneurons in lumbar spinal cord of the frog, rana catesbiana. J. Neurophysiol. 37, 691–704. 10.1152/jn.1974.37.4.691 4366213

[B16] d'Avella A. Bizzi E. (2005). Shared and specific muscle synergies in natural motor behaviors. Proc. Nat. Acad. Sci. 102, 3076–3081. 10.1073/pnas.0500199102 15708969 PMC549495

[B17] d'Avella A. Giese M. Ivanenko Y. P. Schack T. Flash T. (2015). Modularity in Motor Control: From Muscle Synergies to Cognitive Action Representation. 10.3389/fncom.2015.00126 PMC4598477 26500533

[B18] de Rugy A. Loeb G. E. Carroll T. J. (2013). Are muscle synergies useful for neural control? Front. Comput. Neurosci. 7:19. 10.3389/fncom.2013.00019 23519326 PMC3604633

[B19] Delp S. L. Anderson F. C. Arnold A. S. Loan P. Habib A. John C. T.. (2007). Opensim: open-source software to create and analyze dynamic simulations of movement. IEEE Trans. Biomed. Eng. 54, 1940–1950. 10.1109/TBME.2007.901024 18018689

[B20] Diamond A. Holland O. (2014). Reaching control of a full-torso, modelled musculoskeletal robot using muscle synergies emergent under reinforcement learning. Bioinspirat. Biomimet. 9:016015. 10.1088/1748-3182/9/1/016015 24523354

[B21] Dittmer S. King E. J. Maass P. (2019). Singular values for relu layers. IEEE Trans. Neural Netw. Learn. Syst. 31, 3594–3605. 10.1109/TNNLS.2019.2945113 31714239

[B22] Fischer F. Bachinski M. Klar M. Fleig A. Müller J. (2021). Reinforcement learning control of a biomechanical model of the upper extremity. Sci. Rep. 11:14445. 10.1038/s41598-021-93760-1 34262081 PMC8280157

[B23] Gallego J. A. Perich M. G. Naufel S. N. Ethier C. Solla S. A. Miller L. E. (2018). Cortical population activity within a preserved neural manifold underlies multiple motor behaviors. Nat. Commun. 9:4233. 10.1038/s41467-018-06560-z 30315158 PMC6185944

[B24] Giszter S. F. Mussa-Ivaldi F. A. Bizzi E. (1993). Convergent force fields organized in the frog's spinal cord. J. Neurosci. 13, 467–491. 10.1523/JNEUROSCI.13-02-00467.1993 8426224 PMC6576636

[B25] Hagio S. Kouzaki M. (2018). Modularity speeds up motor learning by overcoming mechanical bias in musculoskeletal geometry. J. Royal Soc. Interf. 15:20180249. 10.1098/rsif.2018.0249 30305418 PMC6228487

[B26] Hayashibe M. Shimoda S. (2014). Synergetic motor control paradigm for optimizing energy efficiency of multijoint reaching via tacit learning. Front. Comput. Neurosci. 8:21. 10.3389/fncom.2014.00021 24616695 PMC3937612

[B27] Herbert R. D. Gandevia S. C. (2019). The passive mechanical properties of muscle. J. Appl. Physiol. 126, 1442–1444. 10.1152/japplphysiol.00966.2018 30412027

[B28] Herzog W. (2019). Passive force enhancement in striated muscle. J. Appl. Physiol. 126, 1782–1789. 10.1152/japplphysiol.00676.2018 31070958 PMC6620658

[B29] Hilt P. M. Delis I. Pozzo T. Berret B. (2018). Space-by-time modular decomposition effectively describes whole-body muscle activity during upright reaching in various directions. Front. Comput. Neurosci. 12:20. 10.3389/fncom.2018.00020 29666576 PMC5891645

[B30] Hirashima M. Nozaki D. (2012). Learning with slight forgetting optimizes sensorimotor transformation in redundant motor systems. PLoS Comput. Biol. 8:e1002590. 10.1371/journal.pcbi.1002590 22761568 PMC3386159

[B31] Hirashima M. Oya T. (2016). How does the brain solve muscle redundancy? Filling the gap between optimization and muscle synergy hypotheses. Neurosci. Res. 104, 80–87. 10.1016/j.neures.2015.12.008 26724372

[B32] Inglis J. T. Horak F. B. Shupert C. L. Jones-Rycewicz C. (1994). The importance of somatosensory information in triggering and scaling automatic postural responses in humans. Exp. Brain Res. 101, 159–164. 10.1007/BF00243226 7843295

[B33] Inouye J. M. Valero-Cuevas F. J. (2016). Muscle synergies heavily influence the neural control of arm endpoint stiffness and energy consumption. PLoS Comput. Biol. 12:e1004737. 10.1371/journal.pcbi.1004737 26867014 PMC4750997

[B34] Isa T. Ohki Y. Alstermark B. Pettersson L.-G. Sasaki S. (2007). Direct and indirect cortico-motoneuronal pathways and control of hand/arm movements. Physiology 22, 145–152. 10.1152/physiol.00045.2006 17420305

[B35] Kargo W. J. Ramakrishnan A. Hart C. B. Rome L. C. Giszter S. F. (2010). A simple experimentally based model using proprioceptive regulation of motor primitives captures adjusted trajectory formation in spinal frogs. J. Neurophysiol. 103, 573–590. 10.1152/jn.01054.2007 19657082 PMC2807239

[B36] Kutsuzawa K. Hayashibe M. (2022). Motor synergy generalization framework for new targets in multi-planar and multi-directional reaching task. R. Soc. Open Sci. 9:211721. 10.1098/rsos.211721 35620009 PMC9114934

[B37] Leonardis J. M. Alkayyali A. A. Lipps D. B. (2020). Posture-dependent neuromuscular contributions to three-dimensional isometric shoulder torque generation. J. Neurophysiol. 123, 1526–1535. 10.1152/jn.00702.2019 32101487

[B38] Loeb G. E. (1983). Finding common groud between robotics and physiology. Trends Neurosci. 6, 203–204. 10.1016/0166-2236(83)90093-0

[B39] Loeb G. E. (2021). Learning to use muscles. J. Hum. Kinet. 76, 9–33. 10.2478/hukin-2020-0084 33603922 PMC7877274

[B40] McKay J. L. Ting L. H. (2008). Functional muscle synergies constrain force production during postural tasks. J. Biomech. 41, 299–306. 10.1016/j.jbiomech.2007.09.012 17980370 PMC4350792

[B41] McKay J. L. Ting L. H. (2012). Optimization of muscle activity for task-level goals predicts complex changes in limb forces across biomechanical contexts. PLoS Comput. Biol. 8:e1002465. 10.1371/journal.pcbi.1002465 22511857 PMC3325175

[B42] Overduin S. A. dAvella A. Carmena J. M. Bizzi E. (2012). Microstimulation activates a handful of muscle synergies. Neuron 76, 1071–1077. 10.1016/j.neuron.2012.10.018 23259944 PMC3547640

[B43] Overduin S. A. d'Avella A. Roh J. Bizzi E. (2008). Modulation of muscle synergy recruitment in primate grasping. J. Neurosci. 28, 880–892. 10.1523/JNEUROSCI.2869-07.2008 18216196 PMC6671000

[B44] Overduin S. A. d'Avella A. Roh J. Carmena J. M. Bizzi E. (2015). Representation of muscle synergies in the primate brain. J. Neurosci. 35, 12615–12624. 10.1523/JNEUROSCI.4302-14.2015 26377453 PMC4571600

[B45] Pham K. Portilla-Jiménez M. Roh J. (2023). Generalizability of muscle synergies in isometric force generation versus point-to-point reaching in the human upper extremity workspace. Front. Hum. Neurosci. 17:1144860. 10.3389/fnhum.2023.1144860 37529403 PMC10387555

[B46] Roberts T. J. (2016). Contribution of elastic tissues to the mechanics and energetics of muscle function during movement. J. Exp. Biol. 219, 266–275. 10.1242/jeb.124446 26792339 PMC6514471

[B47] Rodriguez K. L. Roemmich R. T. Cam B. Fregly B. J. Hass C. J. (2013). Persons with parkinson disease exhibit decreased neuromuscular complexity during gait. Clini. Neurophysiol. 124, 1390–1397. 10.1016/j.clinph.2013.02.006 23474055 PMC3679225

[B48] Roh J. Cheung V. C. Bizzi E. (2011). Modules in the brain stem and spinal cord underlying motor behaviors. J. Neurophysiol. 106, 1363–1378. 10.1152/jn.00842.2010 21653716 PMC3174810

[B49] Roh J. Rymer W. Z. Beer R. F. (2012). Robustness of muscle synergies underlying three-dimensional force generation at the hand in healthy humans. J. Neurophysiol. 107, 2123–2142. 10.1152/jn.00173.2011 22279190 PMC3331600

[B50] Rokni U. Richardson A. G. Bizzi E. Seung H. S. (2007). Motor learning with unstable neural representations. Neuron 54, 653–666. 10.1016/j.neuron.2007.04.030 17521576

[B51] Rückert E. d'Avella A. (2013). Learned parametrized dynamic movement primitives with shared synergies for controlling robotic and musculoskeletal systems. Front. Comput. Neurosci. 7:138. 10.3389/fncom.2013.00138 24146647 PMC3797962

[B52] Sartori M. Gizzi L. Lloyd D. G. Farina D. (2013). A musculoskeletal model of human locomotion driven by a low dimensional set of impulsive excitation primitives. Front. Comput. Neurosci. 7:79. 10.3389/fncom.2013.00079 23805099 PMC3693080

[B53] Saul K. R. Hu X. Goehler C. M. Vidt M. E. Daly M. Velisar A.. (2015). Benchmarking of dynamic simulation predictions in two software platforms using an upper limb musculoskeletal model. Comput. Methods Biomech. Biomed. Engin. 18, 1445–1458. 10.1080/10255842.2014.916698 24995410 PMC4282829

[B54] Scott S. H. (2008). Inconvenient truths about neural processing in primary motor cortex. J. Physiol. 586, 1217–1224. 10.1113/jphysiol.2007.146068 18187462 PMC2375659

[B55] Sharif Razavian R. Mehrabi N. McPhee J. (2015). A model-based approach to predict muscle synergies using optimization: application to feedback control. Front. Comput. Neurosci. 9:121. 10.3389/fncom.2015.00121 26500530 PMC4593861

[B56] Shenoy K. V. Sahani M. Churchland M. M. (2013). Cortical control of arm movements: a dynamical systems perspective. Annu. Rev. Neurosci. 36, 337–359. 10.1146/annurev-neuro-062111-150509 23725001

[B57] Sohn M. H. Ting L. H. (2016). Suboptimal muscle synergy activation patterns generalize their motor function across postures. Front. Comput. Neurosci. 10:7. 10.3389/fncom.2016.00007 26869914 PMC4740401

[B58] Song Y. Hirashima M. Takei T. (2022). Neural network models for spinal implementation of muscle synergies. Front. Syst. Neurosci. 16:800628. 10.3389/fnsys.2022.800628 35370571 PMC8965765

[B59] Stapley P. J. Ting L. H. Hulliger M. Macpherson J. M. (2002). Automatic postural responses are delayed by pyridoxine-induced somatosensory loss. J. Neurosci. 22, 5803–5807. 10.1523/JNEUROSCI.22-14-05803.2002 12122040 PMC6757909

[B60] Sternad D. Marino H. Charles S. K. Duarte M. Dipietro L. Hogan N. (2013). Transitions between discrete and rhythmic primitives in a unimanual task. Front. Comput. Neurosci. 7:90. 10.3389/fncom.2013.00090 23888139 PMC3719015

[B61] Sy H. V. N. Nambu I. Wada Y. (2016). The adjustment of muscle synergy recruitment by controlling muscle contraction during the reaching movement, in 2016 IEEE International Conference on Systems, Man, and Cybernetics (SMC) (Budapest: IEEE), 000756–000761.

[B62] Taïx M. Tran M. T. Souères P. Guigon E. (2013). Generating human-like reaching movements with a humanoid robot: a computational approach. J. Comput. Sci. 4:269–284. 10.1016/j.jocs.2012.08.001

[B63] Takei T. Confais J. Tomatsu S. Oya T. Seki K. (2017). Neural basis for hand muscle synergies in the primate spinal cord. Proc. Nat. Acad. Sci. 114:8643–8648. 10.1073/pnas.1704328114 28739958 PMC5559022

[B64] Takiyama K. Okada M. (2012). Maximization of learning speed in the motor cortex due to neuronal redundancy. PLoS Comput. Biol. 8:e1002348. 10.1371/journal.pcbi.1002348 22253586 PMC3257280

[B65] Ting L. H. Chiel H. J. Trumbower R. D. Allen J. L. McKay J. L. Hackney M. E.. (2015). Neuromechanical principles underlying movement modularity and their implications for rehabilitation. Neuron 86, 38–54. 10.1016/j.neuron.2015.02.042 25856485 PMC4392340

[B66] Ting L. H. Macpherson J. M. (2005). A limited set of muscle synergies for force control during a postural task. J. Neurophysiol. 93, 609–613. 10.1152/jn.00681.2004 15342720

[B67] Torres-Oviedo G. Macpherson J. M. Ting L. H. (2006). Muscle synergy organization is robust across a variety of postural perturbations. J. Neurophysiol. 96, 1530–1546. 10.1152/jn.00810.2005 16775203

[B68] Winters J. Stark L. Seif-Naraghi A.-H. (1988). An analysis of the sources of musculoskeletal system impedance. J. Biomech. 21, 1011–1025. 10.1016/0021-9290(88)90248-5 2577948

[B69] Yang Q. Logan D. Giszter S. F. (2019). Motor primitives are determined in early development and are then robustly conserved into adulthood. Proc. Nat. Acad. Sci. 116, 12025–12034. 10.1073/pnas.1821455116 31138689 PMC6575561

[B70] Yaron A. Kowalski D. Yaguchi H. Takei T. Seki K. (2020). Forelimb force direction and magnitude independently controlled by spinal modules in the macaque. Proc. Nat. Acad. Sci. 117, 27655–27666. 10.1073/pnas.1919253117 33060294 PMC7959559

